# Ovarian non-gestational placental site trophoblastic tumor with lung metastasis: further evidence for a distinct category of trophoblastic neoplasm

**DOI:** 10.1186/s13000-023-01436-3

**Published:** 2024-01-03

**Authors:** Maryam Shahi, Levon Katsakhyan, Mark Hopkins, Wendy Allen-Rhoades, Marissa K. Cepress, Carrie Langstraat, Michael B. Ishitani, Russell Vang, Brigitte M. Ronnett, Deyin Xing

**Affiliations:** 1 Department of Laboratory Medicine, Mayo Clinic, Rochester, MN, USA; 2 Department of Pediatrics, Mayo Clinic, Rochester, MN, USA; 3 Department of Obstetrics and Gynecology, Mayo Clinic, Rochester, MN, USA; 4 Department of Pediatric Surgery, Mayo Clinic, Rochester, MN, USA; 5 Department of Pathology, The Johns Hopkins Medical Institutions, Baltimore, MD USA; 6 Department of Gynecology and Obstetrics, The Johns Hopkins Medical Institutions, Baltimore, MD USA; 7 Department of Oncology, The Johns Hopkins Medical Institutions, Baltimore, MD USA

**Keywords:** Molecular genotyping, Non-gestational trophoblastic tumor, Ovarian germ cell tumor, Placental site trophoblastic tumor

## Abstract

We previously described a series of cases which characterize a distinct group of primary ovarian placental site trophoblastic tumor (PSTT) and epithelioid trophoblastic tumor (ETT) as a non-gestational set consistent with germ cell type/origin. Here we report a new case of ovarian non-gestational PSTT. The patient was a 13 year-old young female admitted for a spontaneous pneumothorax of the left lung. The pathology of lung wedge excision specimen demonstrated metastatic PSTT and ovarian biopsy showed atypical intermediate trophoblastic proliferation which was found to be PSTT in the subsequent salpingo-oophorectomy specimen. In the ovary, the tumor was composed of singly dispersed or small clusters of predominantly mononuclear cells and rare multinucleated cells extensively infiltrating the ovarian parenchyma, tubal mucosa, and paraovarian/paratubal soft tissue. A minor component of mature cystic teratoma (less than 5% of total tumor volume) was present. Immunohistochemically, the neoplastic cells of main tumor were diffusely immunoreactive for hPL, Gata3 and AE1/AE3, and had only rare hCG-positive or p63-positive cells. The morphology and immunohistochemical results support a PSTT. Molecular genotyping revealed an identical genotype pattern between the normal lung tissue and the metastatic PSTT, indicating its non-gestational nature of germ cell type/origin. This case represents the first case of such tumor with distant (lung) metastasis. This case also provides further evidence to support our recommendation that primary ovarian non-gestational intermediate trophoblastic tumors of germ cell type/origin, including PSTT and ETT, should be formally recognized in classification systems.

## Introduction

Placental site trophoblastic tumor (PSTT) is an uncommon trophoblastic neoplasm consisting of neoplastic implantation site-type intermediate trophoblast (IT) which is thought to be derived from tumorigenic transformation of cytotrophoblast [1]. Different from choriocarcinoma and epithelioid trophoblastic tumor (ETT), PSTT usually lacks the biphasic or trimorphic pattern of proliferation seen in the former and epithelioid morphology in the latter [2–4]. Unlike choriocarcinoma which is often associated with a complete hydatidiform mole, two thirds of PSTT cases follow a full term pregnancy [5]. Clinically, while most patients are cured by simple hysterectomy, 25–30% of tumors may behave aggressively and develop recurrent disease [6].

In the 2014 and 2020 WHO classification of female genital tumors, PSTT and ETT are only formally recognized as gestational trophoblastic neoplasms [3, 4]. We previously described a series of cases which characterize a distinct group of primary ovarian PSTT and ETT as a non-gestational set consistent with germ cell type/origin [7]. Although extremely rare but given their unique genetics, biology and pathogenesis, we propose that this category of trophoblastic neoplasm, i.e., non-gestational PSTT and ETT, should be recognized in female genital tumor classification systems, similar to their counterparts in the testis [8–10]. Here, we report a new case of ovarian non-gestational PSTT associated with mature cystic teratoma, which provides further evidence to support our recommendation. This case also represents the first case of such tumor with distant (lung) metastasis, highlighting its malignant metastatic potential.

## Case study

### Clinical information

**Fig. 1:**
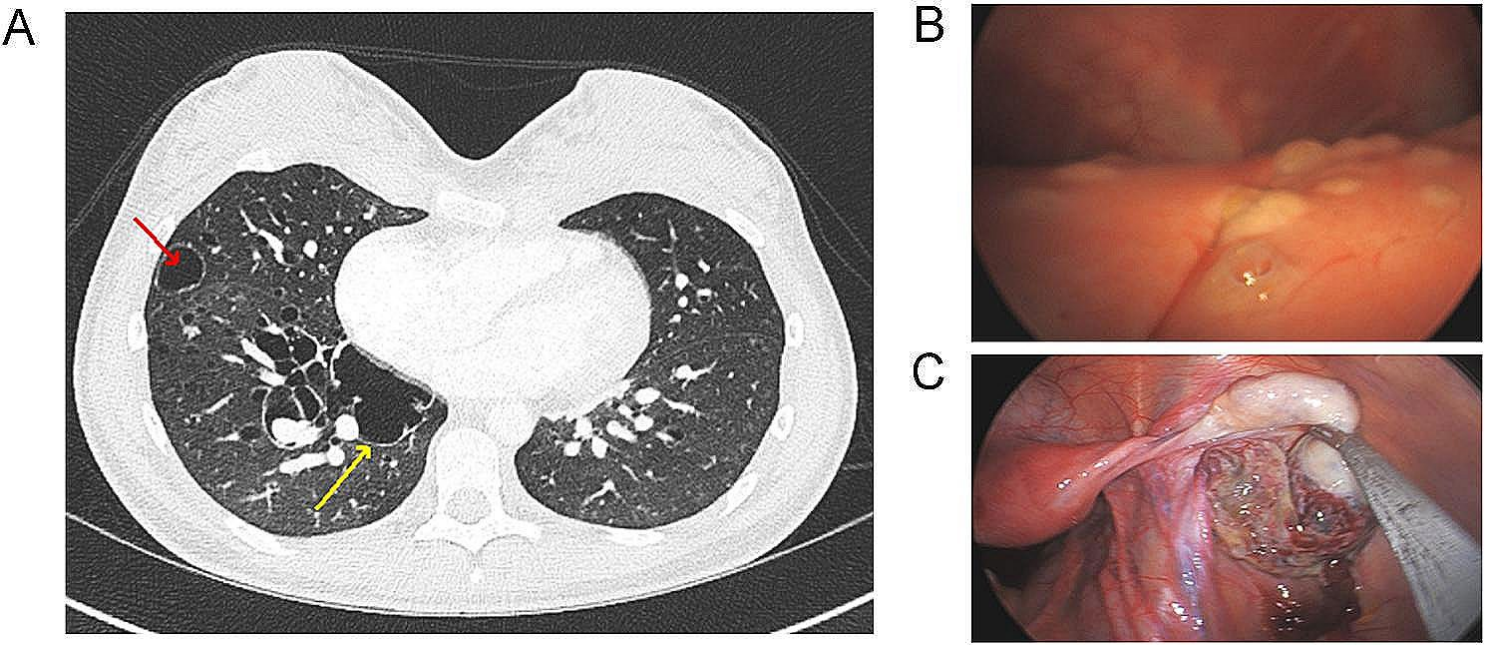
Computerized tomography (CT) scan identified multiple thin-walled cysts throughout both lungs (**A**, arrows indicate cysts). Thoracoscopic examination showed that the lungs surface was notable for multiple cystic lesions and nodules (**B**). Laparoscopy examination demonstrated that the right ovary and tube appeared inflamed with attached partially necrotic hemorrhagic cyst (**C**)

The 13 year-old young female patient initially presented to her local healthcare facility with shortness of breath, nausea and vomiting, and headache. Other symptoms included deepening voice, mild clitoromegaly, and cystic acne. Chest x-ray demonstrated a large left tension pneumothorax. During this hospitalization, computerized tomography (CT) scan of chest abdomen pelvis with contrast was performed and identified multiple/diffuse cystic changes involving all lobes of the lung (Fig. 1A), multiple pulmonary artery aneurysms-pseudoaneurysms and potential adnexal mass. The patient had elevated levels of CA19-9 and CA-125 but these markers decreased to normal during her hospital stay. At the presentation, she had elevated serum β-hCG (194 mIU/mL, normal level <2mIU/mL ) and increased testosterone (413 ng/dL, normal level for age/gender <31.5 ng/dl). She underwent a video-assisted thoracic surgery (VATS) for right middle lobe and lower lobe wedge excision as well as a laparoscopy with ovarian biopsy. Thoracoscopic examination showed that the lungs surface was notable for multiple cystic lesions and nodules (Fig. 1B). Laparoscopy examination demonstrated that the right ovary and tube appeared inflamed with attached partially necrotic hemorrhagic cyst (Fig. 1C). The pathology of lung specimen demonstrated metastatic PSTT and ovarian biopsy showed atypical intermediate trophoblastic proliferation due to very limited amount of lesional tissue. Subsequently she underwent right salpingo-oophorectomy which showed a PSTT with minor component of mature cystic teratoma. After the procedure, the patient still has consistently elevated hCG (most recent hCG 206 mIU/mL, one month after oophorectomy) and she is currently on chemotherapy.

### Gross and histopathological findings

**Fig. 2:**
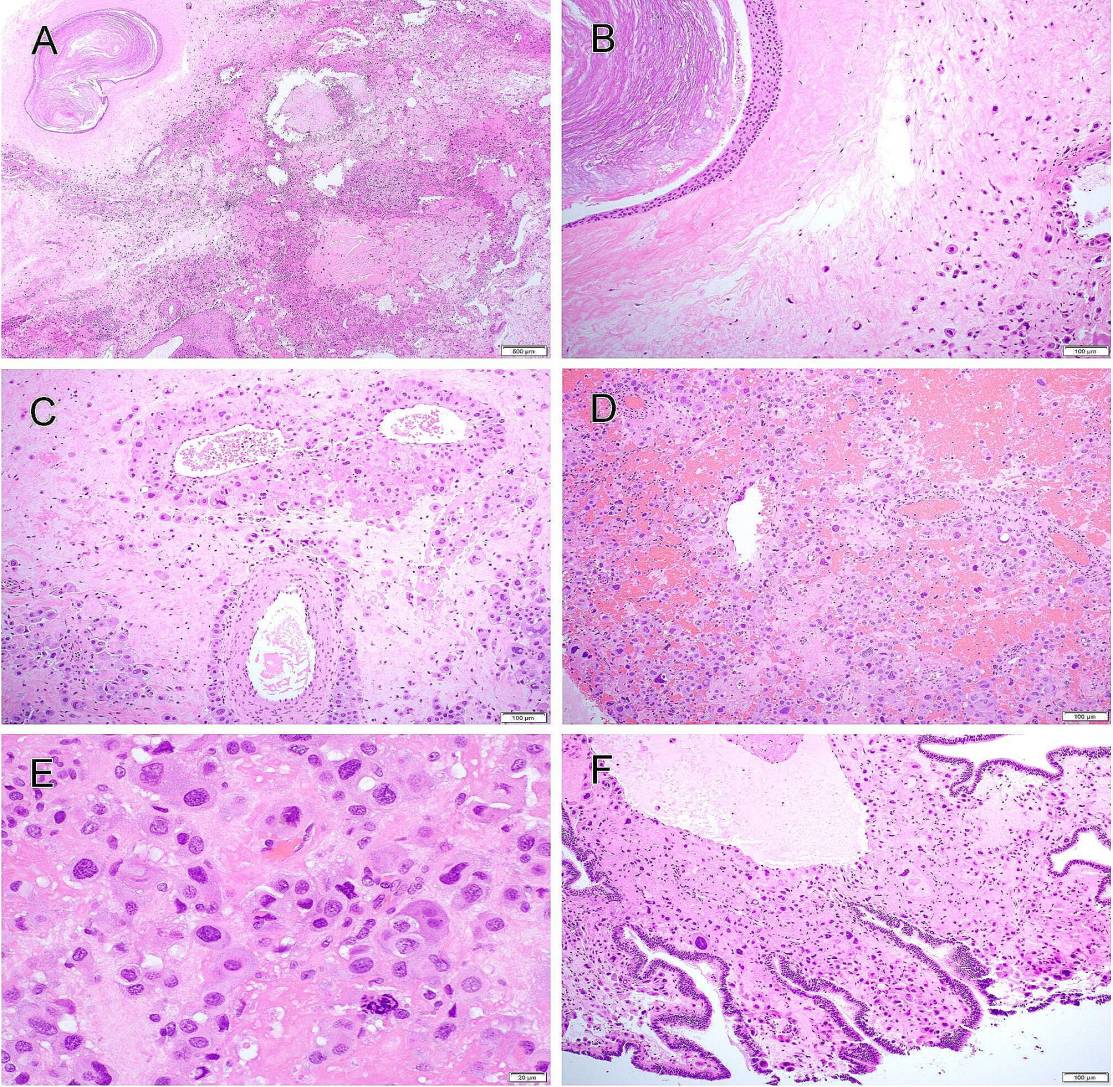
Non-gestational placental site trophoblastic tumor (PSTT) in the ovary. Section demonstrated a mass lesion with solid and cystic growth pattern and hemorrhage (**A**). The tumor was composed of singly dispersed predominantly mononuclear cells extensively infiltrating the ovarian parenchyma (**B**). A minor component of mature cystic teratoma was present (**B**). The tumor cells displayed perivascular accentuation around the arteries and replacement of the vascular wall of veins (**C**). The hemorrhagic areas contained loosely cohesive tumor cells (**D**). On high power, the polygonal tumor cells showed irregular and hyperchromatic nuclei, occasional nuclear pseudoinclusions, and abundant eosinophilic to amphophilic cytoplasm (**E**). The tumor diffusely involved fallopian tube mucosa (**F**)

The right salpingo-oophorectomy specimen revealed an ovary measuring 6.0 × 3.0 × 2.5 cm and an attached fallopian tube measuring 10.0 × 0.6 cm with hemorrhagic fimbriae. Ovarian sectioning showed a 3.8 × 3.0 × 1.8 cm area of hemorrhage while the reminder had a solid cut surface. Histological sections demonstrated a mass lesion with solid and cystic growth pattern and hemorrhage (Fig. 2A). The tumor was composed of singly dispersed or small clusters of predominantly mononuclear cells and rare multinucleated cells extensively infiltrating the ovarian parenchyma (Fig. 2B). Background ovary showed frequent follicles in various stages as well as small cystic areas of mature teratoma composed of ciliate, mucinous, and squamous epithelium (Fig. 2B) which accounted for less than 5% of total tumor volume. The tumor cells displayed perivascular accentuation around the arteries and replacement of the vascular wall of veins (Fig. 2C). The hemorrhagic areas contained loosely cohesive tumor cells (Fig. 2D). On high power, the polygonal tumor cells showed irregular and hyperchromatic nuclei, occasional nuclear pseudoinclusion, and abundant eosinophilic to amphophilic cytoplasm (Fig. 2E). Rare mitotic figures were noted. The tumor diffusely involved fallopian tube mucosa (Fig. 2F), stroma, and paratubal/paraovarian soft tissue.

**Fig. 3:**
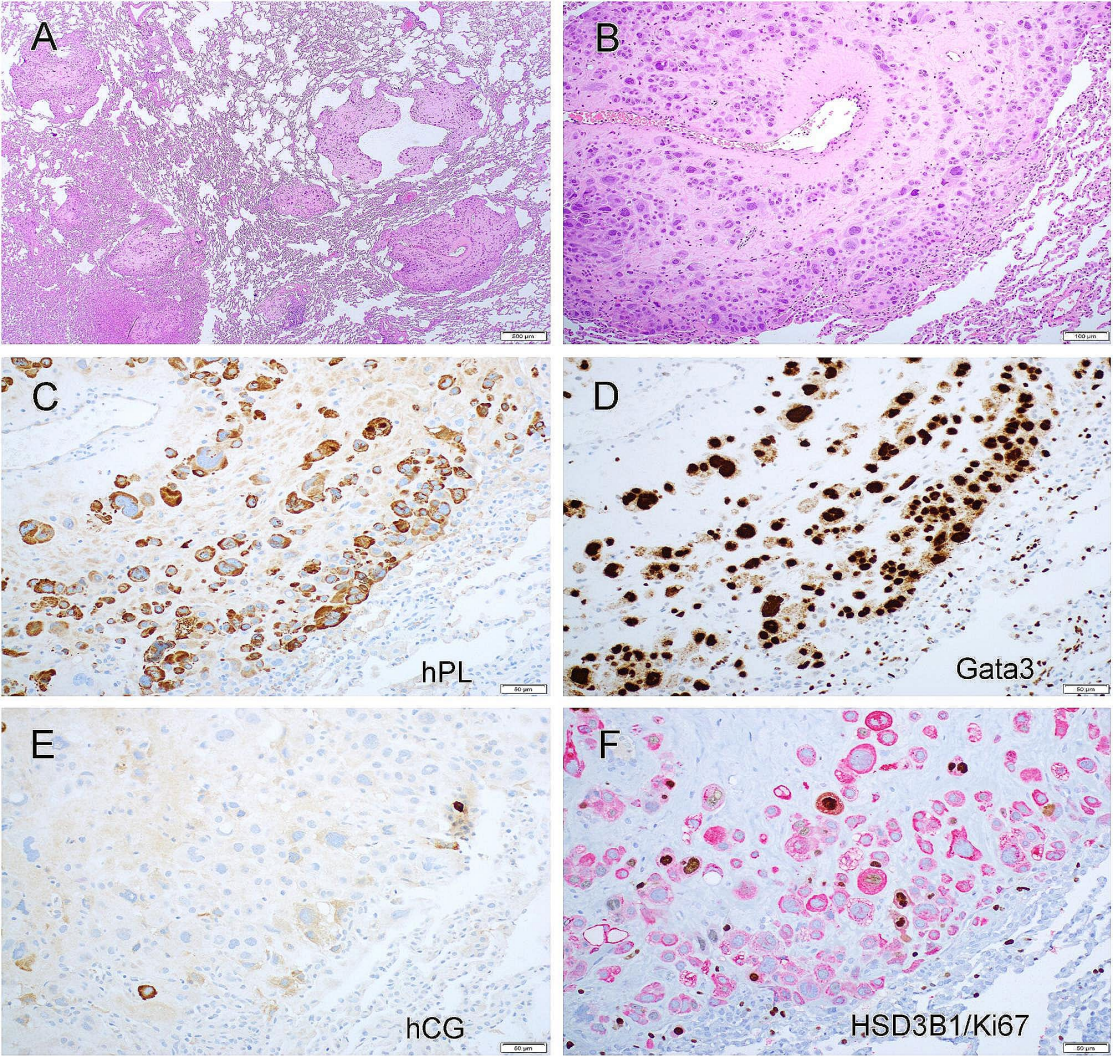
Metastatic placental site trophoblastic tumor (PSTT) in the lung. Section showed multiple metastatic tumor nodules (**A**) in the lung which displayed similar morphology to that of the ovarian lesion (**B**). The tumor cells were diffusely immunoreactive for hPL (**C**) and Gata3 (**D**), and had only rare hCG-positive cells (**E**). HSD3B1/Ki67 double stain showed increased Ki-67 proliferation index in HSD3B1-positive cells (**F**)

Prior to the right salpingo-oophorectomy, a laparoscopic ovarian biopsy and right middle lobe and lower lobe lung wedge excision were performed. The ovarian specimen showed atypical intermediate trophoblastic cell proliferation. Histological examination of lung specimen demonstrated multiple metastatic tumor nodules (Fig. 3A) which displayed similar morphology to that of the ovarian lesion (Fig. 3B).

### Immunohistochemistry and molecular genotyping

Immunohistochemical stains performed on the lung tissue demonstrated that the tumor cells were diffusely immunoreactive for hPL (Fig. 3C), Gata3 (Fig. 3D) and AE1/AE3, and had only rare hCG-positive cells (Fig. 3E) and rare p63-positive cells. HSD3B1/Ki67 double stained showed increased Ki-67 proliferation index in HSD3B1-positive cells (Fig. 3F). The tumor cells were negative for SALL4. The morphology and immunohistochemical results support a metastatic PSTT.

**Fig. 4:**
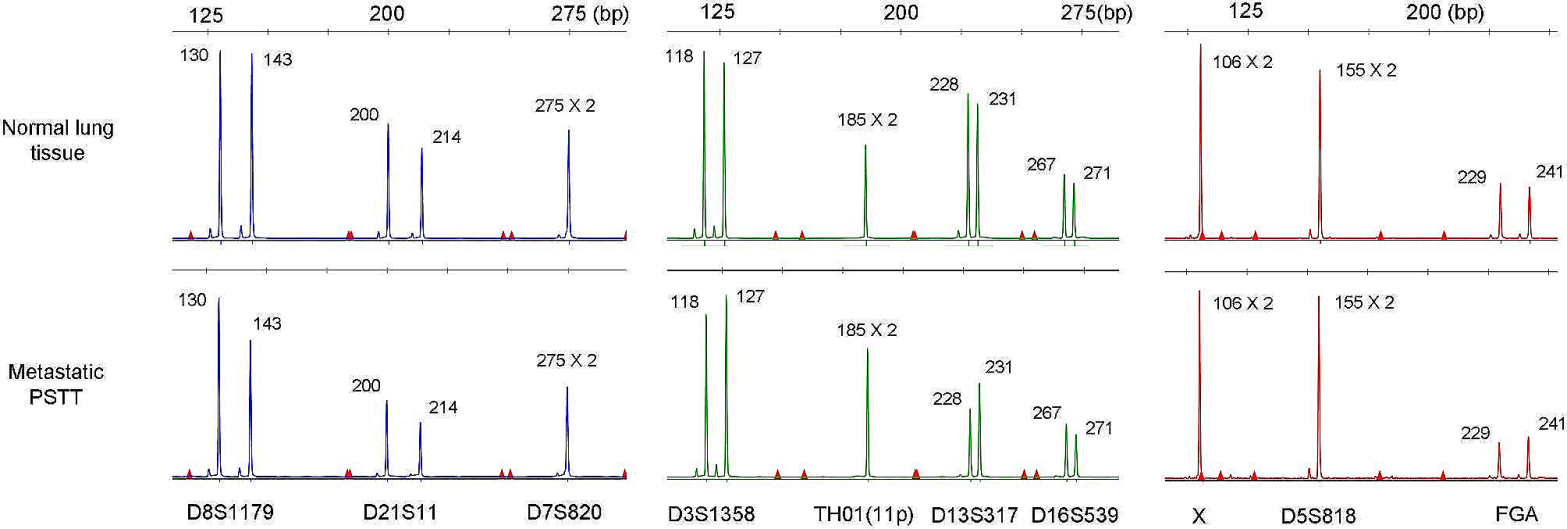
Genotyping demonstrated that the DNA pattern from the metastatic placental site trophoblastic tumor (PSTT) matched that of normal lung tissue, without any novel/unmatched alleles, consistent with a non-gestational tumor

To perform molecular genotyping, genomic DNA was isolated from an area identified as “N” (normal lung tissue) and an area identified as “T” (tumor). The genotype pattern from each tissue was determined and the patterns compared. This assay consists of PCR amplification of fifteen microsatellite markers (CSF1PO, D2S1338, D3S1358, D5S818, D7S820, D8S1179, D13S317, D16S539, D18S51, D19S433, D21S11, FGA, THO1, TPOX, VWA) and the amelogenin locus using AmpFISTR Identifiler PCR amplification kit (Applied Biosystems) [11–13].  The resulting PCR products were analyzed by capillary electrophoresis to identify the alleles for each locus. The genotype pattern was identical between the patient’s normal lung tissue and the metastatic PSTT, without any novel/unmatched alleles (Fig. 4). These results are consistent with a non-gestational tumor which is of germ cell in type/origin.

## Discussion

We have previously described three cases of ovarian non-gestational PSTT of germ cell type/origin [7]. Our current case demonstrated identical genotype patterns between the normal lung tissue and the metastatic PSTT, confirming its non-gestational nature and supporting a germ cell type/origin for this case as well. To the best of our knowledge, the current case represents the fourth published case of this type of tumor and the first case with lung metastasis. Of 4 cases, the mean age of the patients was 22 years (range from 2.5 to 43 years), which is younger than those reported in gestational PSTT (mean 30; range from 20 to 63 years). Tumors ranged in size from 3.0 to 6.0 cm (mean, 4.7; median, 4.8). All tumors displayed classical morphologic features of PSTT which is composed of polygonal implantation site-type IT cells with eosinophilic to amphophilic cytoplasm and irregular, hyperchromatic nuclei. Immunohistochemically, all tumors diffusely expressed hPL with scattered hCG- and p63-positive cells. The combined morphology and immunoprofile support interpretation as PSTT and exclude the possibility of choriocarcinoma or ETT. In the uterus, the PSTT cells often characteristically separate muscle bundles when invading the myometrium and tend to surround and invade blood vessels, extending into the vascular lumen. Likewise, the tumor in the current case extensively involved ovarian parenchyma, infiltrated into tubal mucosa and paratubal/paraovarian soft tissue, and invaded blood vessels with vascular wall replacement, highlighting its similar phenotype and behavior in the extrauterine location.

PSTT commonly develops after a normal pregnancy but may follow hydatidiform moles and spontaneous abortions [14]. Because the latency for tumor development can be up to 20 years, the relationship between PSTT and previous gestation is somewhat uncertain. This situation becomes more complicated when PSTT is found in extrauterine sites. Extrauterine PSTTs have been reported in multiple locations including ovary [15, 16], fallopian tube [15, 17, 18], pelvic wall [19], vagina [20], pouch of Douglas [21], and lung [22, 23]. Traditionally classified as a gestational trophoblastic neoplasm, extrauterine PSTT is thought to arise from an ectopic pregnancy or as a metastasis from a uterine PSTT [15]. Interestingly, similar to our three previously reported non-gestational PSTTs, the tumor in our current case also coexisted with mature cystic teratoma in the ovary, indicating a common germ cell origin [7]. While our patient is of reproductive age (menarche at age 11) and a gestational PSTT is possible, the patient-reported absence of sexual activity and the genotyping data excluded the possibility of a gestational tumor. Of note, one of our previous cases was a 30-month-old girl with a PSTT coexisting with mature cystic teratoma, demonstrating a clinical scenario in which a gestational tumor is not possible and supporting that PSTT in prepubescent individuals must be a form of gonadal germ cell tumor, even if a teratomatous component is not identified [7, 16].

In the absence of ancillary molecular analysis a non-gestational trophoblastic tumor can be diagnosed in children, but in women of reproductive age it is difficult to determine with certainty whether an extrauterine PSTT is gestational or non-gestational based on morphology alone. Given that non-gestational PSTT is so rare, an extra-uterine PSTT in a woman of reproductive age will most likely be assumed to be a gestational tumor and attributed to one of the following scenarios: (1) metastatic from an undetected/occult uterine tumor or (2) related to an ectopic gestational event when encountered in other sites such as ovary and fallopian tube if the uterus is negative. While the finding of a conventional germ cell component such as mature teratoma can provide evidence to suggest a non-gestational PSTT of germ cell type/origin, this component might not be detected in all cases as a result of being grossly non-evident and unsampled or overgrown. In such a situation, gestational origin will likely be assumed, as indicated in the above scenarios. Of note, the teratoma component was limited and fortuitously sampled in all 3 of our previous cases, indicating that thorough sampling is advisable when PSTT is encountered in the ovary in the absence of a uterine tumor. Whether teratoma is identified or not, we advocate genotyping for definitive determination of the nature (gestational versus non-gestational) of a PSTT in the ovary, as well as for trophoblastic tumors in extra-uterine sites in general or when encountered with other neoplastic components regardless of site (uterus, ovary, extra-genital tract) [24–26]. It has been demonstrated that gestational and non-gestational choriocarcinoma have significant differences in prognosis and require different therapy [27]. While there is insufficient data to know if, similarly, the usual gestational PSTT and the rare non-gestational PSTT also have different behavior and require different therapeutic approaches, this potential exists for these genetically distinct entities. Prospective genotyping of all such cases will allow for accumulation of data regarding differences in behavior and response to therapy, which will provide prognostic data and may prove useful for guiding modified therapeutic approaches. In addition, determination of the gestational versus non-gestational nature of a trophoblastic tumor via genotyping might have ethical/legal implications for certain clinical scenarios (such as in patients who are post-menarcheal yet underage).

As we discussed previously, the data regarding non-gestational PSTT and ETT is too limited to provide meaningful insights into their behavior and prognosis. In our previous study, of 4 cases, one non-gestational PSTT and one non-gestational ETT manifested malignant behavior, illustrated by lymph node metastasis in the former and bowel invasion in the latter [7]. The current case represents the first case of such a tumor with distant lung metastasis, providing evidence that these tumors have the potential to behave in an aggressive fashion.

In our previous study we demonstrated that non-gestational PSTTs can contain either a homozygous or a heterozygous genome [7]. The current case showed a heterozygous/biallelic pattern with allelic balance. We speculate that a premeiotic germ cell or a germ cell with meiosis I failure undergoes transformation towards implantation-site type IT cells which then form a PSTT. In contrast, a homozygous pattern of tumor is thought to arise from a germ cell after having undergone meiosis I, which was seen in one of our previous cases of a mature cystic teratoma and a PSTT [7]. Ideally, mature cystic teratoma in this case should be genotyped in conjunction with PSTT to assess whether these tumors develop at the same stage in the meiotic process. However, the current case had a minor component of teratoma for which a macrodissection could not be performed to extract enough genomic DNA for genotyping.

In summary, we report the first case of ovarian non-gestational PSTT with lung metastasis. Similar to the cases we reported previously, the ovarian tumor in the current case characterizes a molecular genotyping-established non-gestational PSTT associated with a minor component of mature cystic teratoma. While the presence of teratoma supports classification as a variant form of “mixed germ cell tumor” in which one component demonstrates trophoblastic differentiation, the dominance of the PSTT or ETT components in these tumors and their potential to spread locally or distantly as pure trophoblastic tumors supports specifically labeling the trophoblastic component in the diagnosis. When teratoma is identified, germ cell type/origin can be indicated as more likely than a tumor-to-tumor metastasis (metastatic uterine PSTT or ETT to ovarian teratoma) and more likely than coincident ectopic gestational PSTT or ETT and ovarian teratoma. In the absence of demonstrable teratoma, genotyping would be recommended (if feasible) to establish the true nature of the tumor, particularly if the uterus lacks a lesion. These cases provide support for adding a new category of non-gestational intermediate trophoblastic tumors, including PSTT and ETT, to ovarian tumor classification systems. A notation can indicate that these are likely of germ cell type/origin but that somatic type/origin might be possible if pure, without another germ cell component, and likely if a rare other type of somatic tumor component is identified [7, 27, 28].

## Data Availability

No datasets were generated or analysed during the current study.
